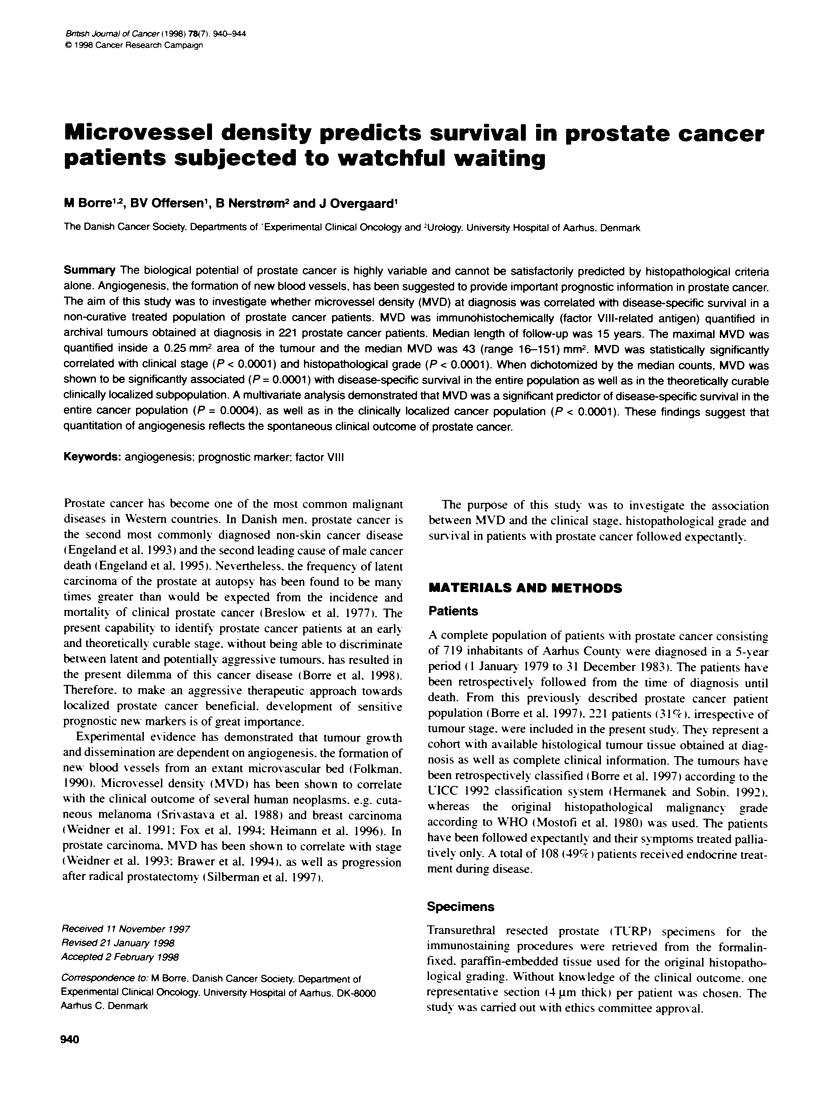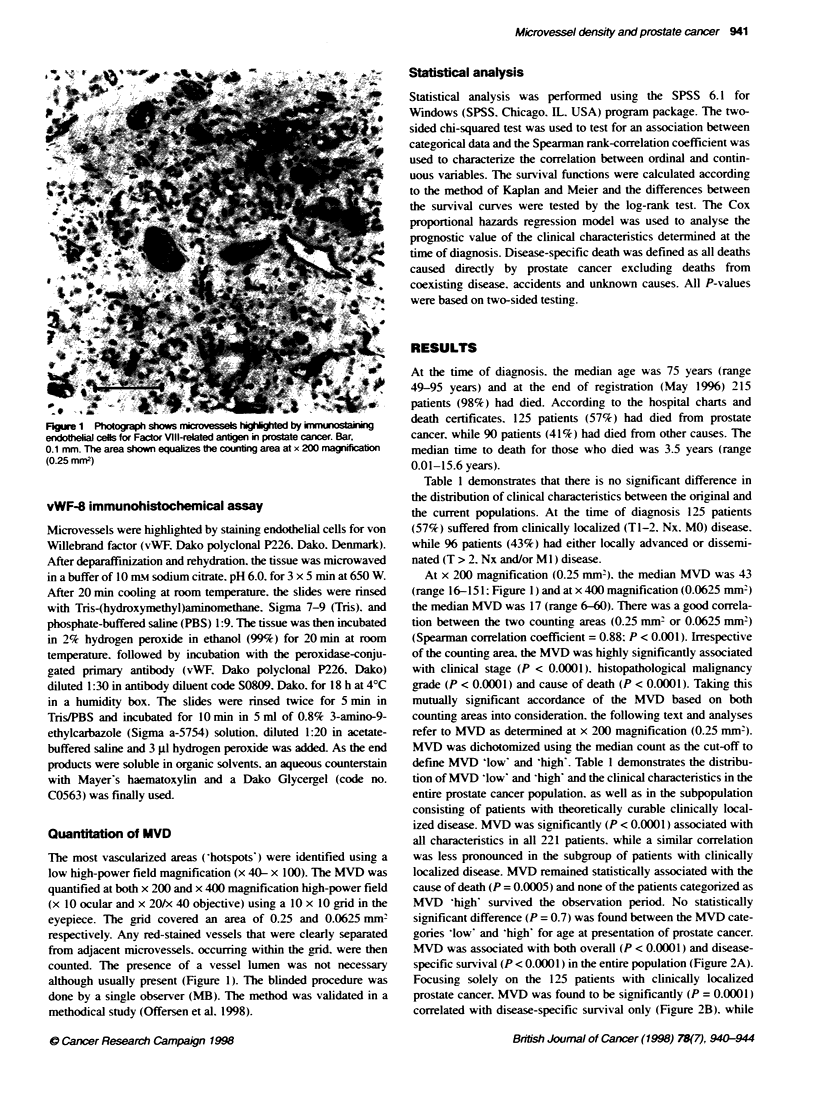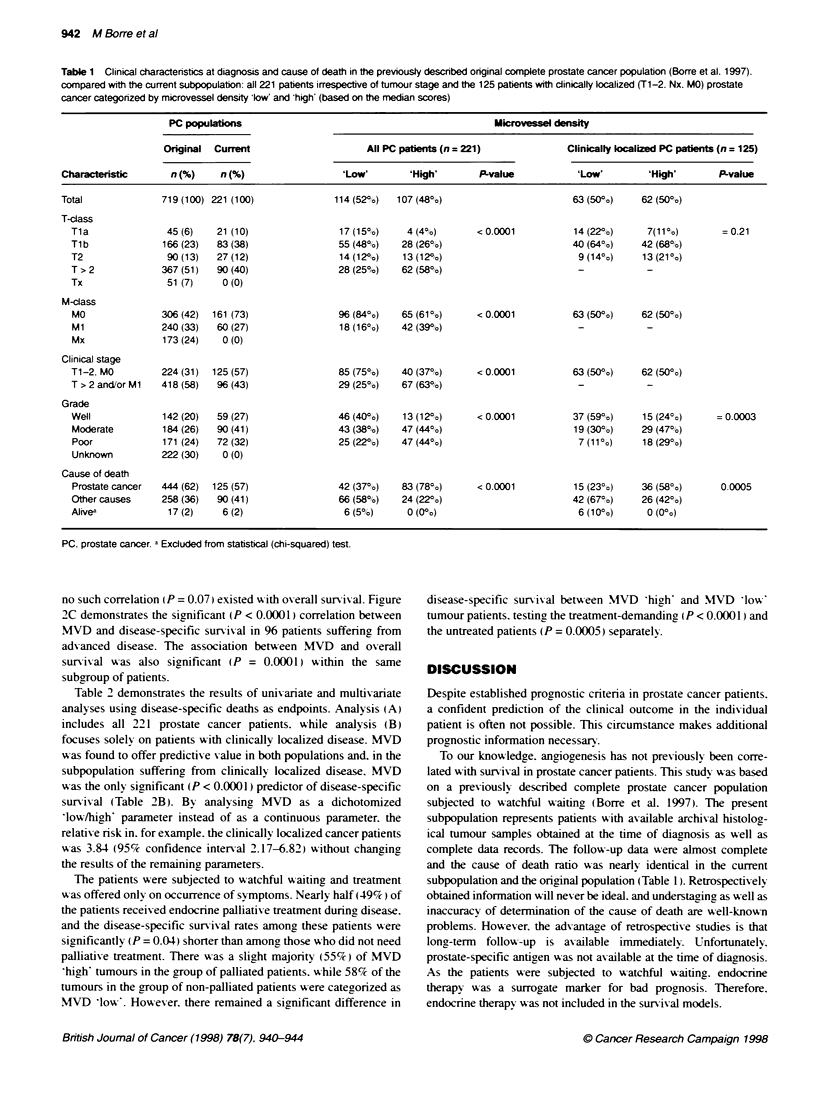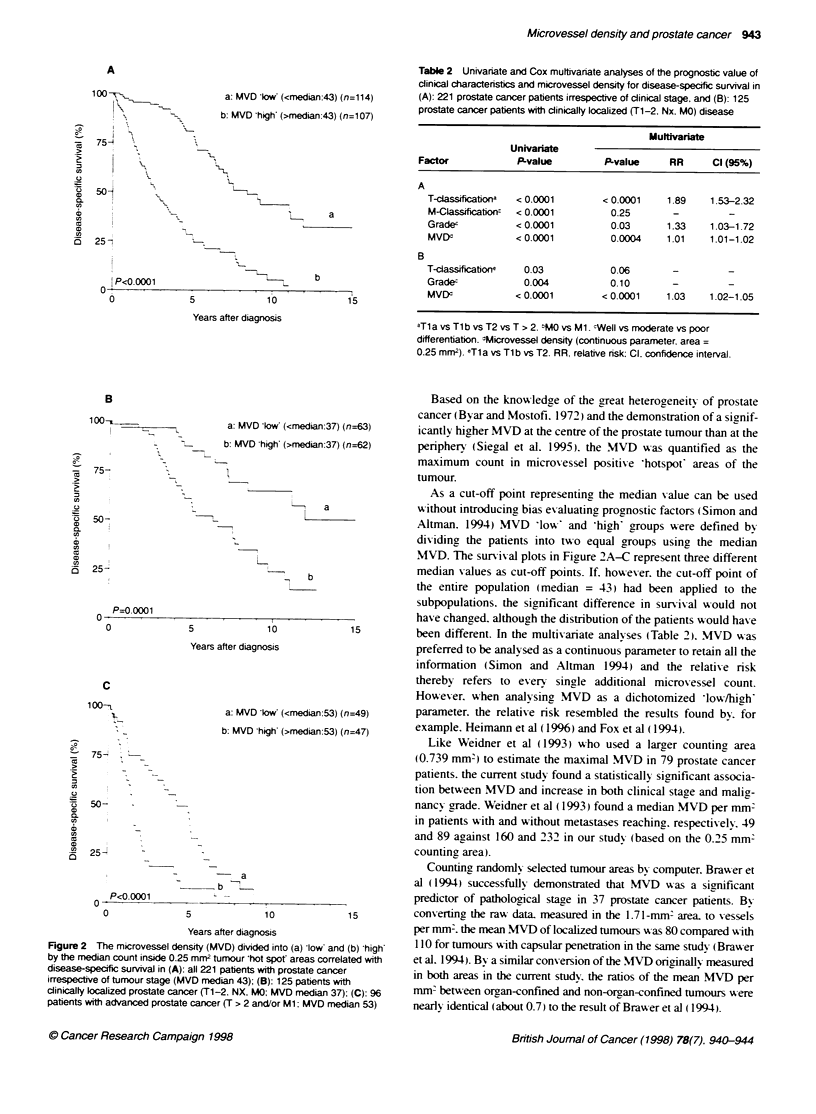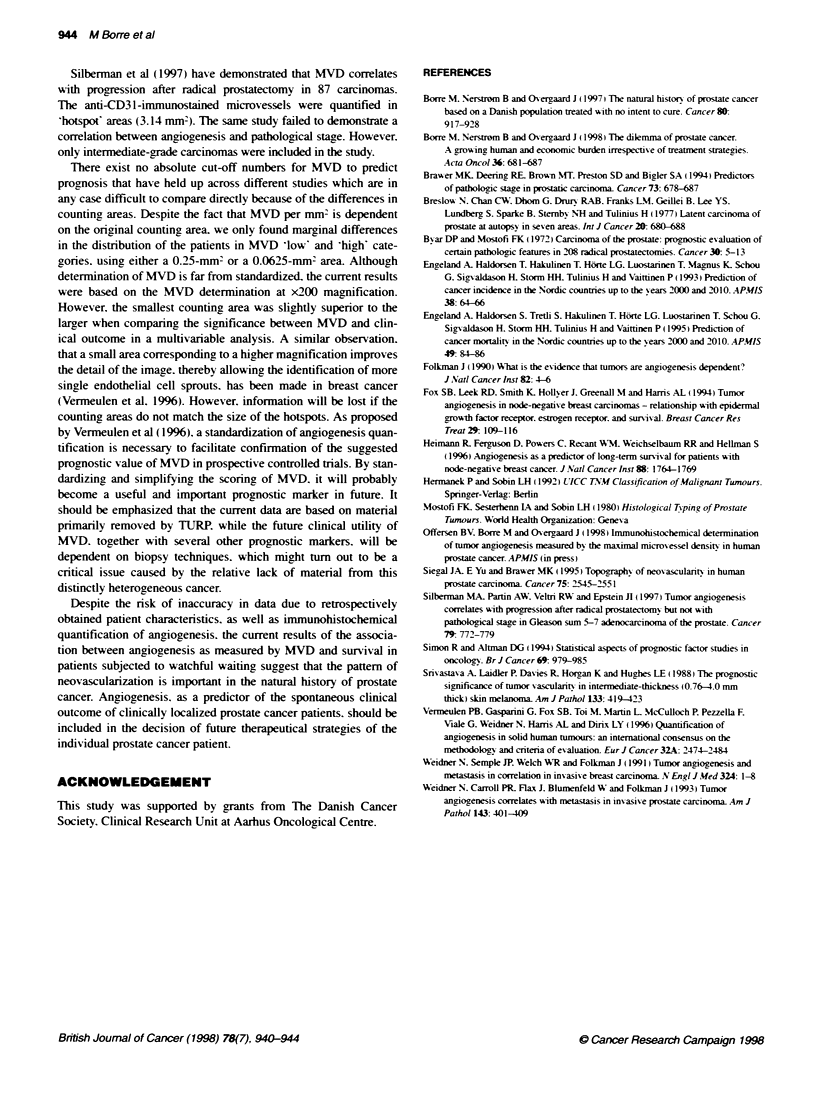# Microvessel density predicts survival in prostate cancer patients subjected to watchful waiting.

**DOI:** 10.1038/bjc.1998.605

**Published:** 1998-10

**Authors:** M. Borre, B. V. Offersen, B. Nerstrøm, J. Overgaard

**Affiliations:** The Danish Cancer Society, Department of Experimental Clinical Oncology, University Hospital of Aarhus.

## Abstract

**Images:**


British Journal of Cancer (1998) 78(7), 940–944
© 1998 Cancer Research Campaign

### Microvessel density predicts survival in prostate cancer patients subjected to watchful waiting

**M Borre[1,2], BV Offersen[1], B Nerstrøm[2] and J Overgaard[1]**

The Danish Cancer Society, Departments of [1]Experimental Clinical Oncology and [2]Urology, University Hospital of Aarhus, Denmark

**Summary** The biological potential of prostate cancer is highly variable and cannot be satisfactorily predicted by histopathological criteria alone. Angiogenesis, the formation of new blood vessels, has been suggested to provide important prognostic information in prostate cancer. The aim of this study was to investigate whether microvessel density (MVD) at diagnosis was correlated with disease-specific survival in a non-curative treated population of prostate cancer patients. MVD was immunohistochemically (factor VIII-related antigen) quantified in archival tumours obtained at diagnosis in 221 prostate cancer patients. Median length of follow-up was 15 years. The maximal MVD was quantified inside a 0.25 mm² area of the tumour and the median MVD was 43 (range 16–151) mm². MVD was statistically significantly correlated with clinical stage ($P < 0.0001$) and histopathological grade ($P < 0.0001$). When dichotomized by the median counts, MVD was shown to be significantly associated ($P = 0.0001$) with disease-specific survival in the entire population as well as in the theoretically curable clinically localized subpopulation. A multivariate analysis demonstrated that MVD was a significant predictor of disease-specific survival in the entire cancer population ($P = 0.0004$), as well as in the clinically localized cancer population ($P < 0.0001$). These findings suggest that quantitation of angiogenesis reflects the spontaneous clinical outcome of prostate cancer.

**Keywords:** angiogenesis; prognostic marker; factor VIII

Prostate cancer has become one of the most common malignant diseases in Western countries. In Danish men, prostate cancer is the second most commonly diagnosed non-skin cancer disease (Engeland et al, 1993) and the second leading cause of male cancer death (Engeland et al, 1995). Nevertheless, the frequency of latent carcinoma of the prostate at autopsy has been found to be many times greater than would be expected from the incidence and mortality of clinical prostate cancer (Breslow et al, 1977). The present capability to identify prostate cancer patients at an early and theoretically curable stage, without being able to discriminate between latent and potentially aggressive tumours, has resulted in the present dilemma of this cancer disease (Borre et al, 1998). Therefore, to make an aggressive therapeutic approach towards localized prostate cancer beneficial, development of sensitive prognostic new markers is of great importance.

Experimental evidence has demonstrated that tumour growth and dissemination are dependent on angiogenesis, the formation of new blood vessels from an extant microvascular bed (Folkman, 1990). Microvessel density (MVD) has been shown to correlate with the clinical outcome of several human neoplasms, e.g. cutaneous melanoma (Srivastava et al, 1988) and breast carcinoma (Weidner et al, 1991; Fox et al, 1994; Heimann et al, 1996). In prostate carcinoma, MVD has been shown to correlate with stage (Weidner et al, 1993; Brawer et al, 1994), as well as progression after radical prostatectomy (Silberman et al, 1997).

*Received 11 November 1997*
*Revised 21 January 1998*
*Accepted 2 February 1998*

*Correspondence to:* M Borre, Danish Cancer Society, Department of Experimental Clinical Oncology, University Hospital of Aarhus, DK-8000 Aarhus C, Denmark

The purpose of this study was to investigate the association between MVD and the clinical stage, histopathological grade and survival in patients with prostate cancer followed expectantly.

### MATERIALS AND METHODS

### Patients

A complete population of patients with prostate cancer consisting of 719 inhabitants of Aarhus County were diagnosed in a 5-year period (1 January 1979 to 31 December 1983). The patients have been retrospectively followed from the time of diagnosis until death. From this previously described prostate cancer patient population (Borre et al, 1997), 221 patients (31%), irrespective of tumour stage, were included in the present study. They represent a cohort with available histological tumour tissue obtained at diagnosis as well as complete clinical information. The tumours have been retrospectively classified (Borre et al, 1997) according to the UICC 1992 classification system (Hermanek and Sobin, 1992), whereas the original histopathological malignancy grade according to WHO (Mostofi et al, 1980) was used. The patients have been followed expectantly and their symptoms treated palliatively only. A total of 108 (49%) patients received endocrine treatment during disease.

### Specimens

Transurethral resected prostate (TURP) specimens for the immunostaining procedures were retrieved from the formalin-fixed, paraffin-embedded tissue used for the original histopathological grading. Without knowledge of the clinical outcome, one representative section (4 µm thick) per patient was chosen. The study was carried out with ethics committee approval.

**Figure 1** Photograph shows microvessels highlighted by immunostaining endothelial cells for Factor VIII-related antigen in prostate cancer. Bar, 0.1 mm. The area shown equalizes the counting area at × 200 magnification (0.25 mm²)

### vWF-8 immunohistochemical assay

Microvessels were highlighted by staining endothelial cells for von Willebrand factor (vWF, Dako polyclonal P226, Dako, Denmark). After deparaffinization and rehydration, the tissue was microwaved in a buffer of 10 mM sodium citrate, pH 6.0, for 3 × 5 min at 650 W. After 20 min cooling at room temperature, the slides were rinsed with Tris-(hydroxymethyl)aminomethane, Sigma 7–9 (Tris), and phosphate-buffered saline (PBS) 1:9. The tissue was then incubated in 2% hydrogen peroxide in ethanol (99%) for 20 min at room temperature, followed by incubation with the peroxidase-conjugated primary antibody (vWF, Dako polyclonal P226, Dako) diluted 1:30 in antibody diluent code S0809, Dako, for 18 h at 4°C in a humidity box. The slides were rinsed twice for 5 min in Tris/PBS and incubated for 10 min in 5 ml of 0.8% 3-amino-9-ethylcarbazole (Sigma a-5754) solution, diluted 1:20 in acetate-buffered saline and 3 µl hydrogen peroxide was added. As the end products were soluble in organic solvents, an aqueous counterstain with Mayer's haematoxylin and a Dako Glycergel (code no. C0563) was finally used.

### Quantitation of MVD

The most vascularized areas ('hotspots') were identified using a low high-power field magnification (× 40– × 100). The MVD was quantified at both × 200 and × 400 magnification high-power field (× 10 ocular and × 20/× 40 objective) using a 10 × 10 grid in the eyepiece. The grid covered an area of 0.25 and 0.0625 mm² respectively. Any red-stained vessels that were clearly separated from adjacent microvessels, occurring within the grid, were then counted. The presence of a vessel lumen was not necessary although usually present (Figure 1). The blinded procedure was done by a single observer (MB). The method was validated in a methodical study (Offersen et al, 1998).

### Statistical analysis

Statistical analysis was performed using the SPSS 6.1 for Windows (SPSS, Chicago, IL, USA) program package. The two-sided chi-squared test was used to test for an association between categorical data and the Spearman rank-correlation coefficient was used to characterize the correlation between ordinal and continuous variables. The survival functions were calculated according to the method of Kaplan and Meier and the differences between the survival curves were tested by the log-rank test. The Cox proportional hazards regression model was used to analyse the prognostic value of the clinical characteristics determined at the time of diagnosis. Disease-specific death was defined as all deaths caused directly by prostate cancer excluding deaths from coexisting disease, accidents and unknown causes. All *P*-values were based on two-sided testing.

### RESULTS

At the time of diagnosis, the median age was 75 years (range 49–95 years) and at the end of registration (May 1996) 215 patients (98%) had died. According to the hospital charts and death certificates, 125 patients (57%) had died from prostate cancer, while 90 patients (41%) had died from other causes. The median time to death for those who died was 3.5 years (range 0.01–15.6 years).

Table 1 demonstrates that there is no significant difference in the distribution of clinical characteristics between the original and the current populations. At the time of diagnosis 125 patients (57%) suffered from clinically localized (T1–2, Nx, M0) disease, while 96 patients (43%) had either locally advanced or disseminated (T > 2, Nx and/or M1) disease.

At × 200 magnification (0.25 mm²), the median MVD was 43 (range 16–151; Figure 1) and at × 400 magnification (0.0625 mm²) the median MVD was 17 (range 6–60). There was a good correlation between the two counting areas (0.25 mm² or 0.0625 mm²) (Spearman correlation coefficient = 0.88; $P < 0.001$). Irrespective of the counting area, the MVD was highly significantly associated with clinical stage ($P < 0.0001$), histopathological malignancy grade ($P < 0.0001$) and cause of death ($P < 0.0001$). Taking this mutually significant accordance of the MVD based on both counting areas into consideration, the following text and analyses refer to MVD as determined at × 200 magnification (0.25 mm²). MVD was dichotomized using the median count as the cut-off to define MVD 'low' and 'high'. Table 1 demonstrates the distribution of MVD 'low' and 'high' and the clinical characteristics in the entire prostate cancer population, as well as in the subpopulation consisting of patients with theoretically curable clinically localized disease. MVD was significantly ($P < 0.0001$) associated with all characteristics in all 221 patients, while a similar correlation was less pronounced in the subgroup of patients with clinically localized disease. MVD remained statistically associated with the cause of death ($P = 0.0005$) and none of the patients categorized as MVD 'high' survived the observation period. No statistically significant difference ($P = 0.7$) was found between the MVD categories 'low' and 'high' for age at presentation of prostate cancer. MVD was associated with both overall ($P < 0.0001$) and disease-specific survival ($P < 0.0001$) in the entire population (Figure 2A). Focusing solely on the 125 patients with clinically localized prostate cancer, MVD was found to be significantly ($P = 0.0001$) correlated with disease-specific survival only (Figure 2B), while

© *Cancer Research Campaign 1998*

Table 1 Clinical characteristics at diagnosis and cause of death in the previously described original complete prostate cancer population (Borre et al. 1997). compared with the current subpopulation: all 221 patients irrespective of tumour stage and the 125 patients with clinically localized (T1–2. Nx. M0) prostate cancer categorized by microvessel density 'low' and 'high' (based on the median scores)

| | PC populations | | Microvessel density | | | | | |
|---|---|---|---|---|---|---|---|---|
| | Original | Current | All PC patients ($n$ = 221) | | | Clinically localized PC patients ($n$ = 125) | | |
| Characteristic | $n$ (%) | $n$ (%) | 'Low' | 'High' | $P$-value | 'Low' | 'High' | $P$-value |
| Total | 719 (100) | 221 (100) | 114 (52%) | 107 (48%) | | 63 (50%) | 62 (50%) | |
| T-class | | | | | | | | |
| T1a | 45 (6) | 21 (10) | 17 (15%) | 4 (4%) | < 0.0001 | 14 (22%) | 7(11%) | = 0.21 |
| T1b | 166 (23) | 83 (38) | 55 (48%) | 28 (26%) | | 40 (64%) | 42 (68%) | |
| T2 | 90 (13) | 27 (12) | 14 (12%) | 13 (12%) | | 9 (14%) | 13 (21%) | |
| T > 2 | 367 (51) | 90 (40) | 28 (25%) | 62 (58%) | | – | – | |
| Tx | 51 (7) | 0 (0) | | | | | | |
| M-class | | | | | | | | |
| M0 | 306 (42) | 161 (73) | 96 (84%) | 65 (61%) | < 0.0001 | 63 (50%) | 62 (50%) | |
| M1 | 240 (33) | 60 (27) | 18 (16%) | 42 (39%) | | – | – | |
| Mx | 173 (24) | 0 (0) | | | | | | |
| Clinical stage | | | | | | | | |
| T1–2. M0 | 224 (31) | 125 (57) | 85 (75%) | 40 (37%) | < 0.0001 | 63 (50%) | 62 (50%) | |
| T > 2 and/or M1 | 418 (58) | 96 (43) | 29 (25%) | 67 (63%) | | – | – | |
| Grade | | | | | | | | |
| Well | 142 (20) | 59 (27) | 46 (40%) | 13 (12%) | < 0.0001 | 37 (59%) | 15 (24%) | = 0.0003 |
| Moderate | 184 (26) | 90 (41) | 43 (38%) | 47 (44%) | | 19 (30%) | 29 (47%) | |
| Poor | 171 (24) | 72 (32) | 25 (22%) | 47 (44%) | | 7 (11%) | 18 (29%) | |
| Unknown | 222 (30) | 0 (0) | | | | | | |
| Cause of death | | | | | | | | |
| Prostate cancer | 444 (62) | 125 (57) | 42 (37%) | 83 (78%) | < 0.0001 | 15 (23%) | 36 (58%) | 0.0005 |
| Other causes | 258 (36) | 90 (41) | 66 (58%) | 24 (22%) | | 42 (67%) | 26 (42%) | |
| Alive[a] | 17 (2) | 6 (2) | 6 (5%) | 0 (0%) | | 6 (10%) | 0 (0%) | |

PC. prostate cancer. [a] Excluded from statistical (chi-squared) test.

no such correlation ($P$ = 0.07) existed with overall survival. Figure 2C demonstrates the significant ($P$ < 0.0001) correlation between MVD and disease-specific survival in 96 patients suffering from advanced disease. The association between MVD and overall survival was also significant ($P$ = 0.0001) within the same subgroup of patients.

Table 2 demonstrates the results of univariate and multivariate analyses using disease-specific deaths as endpoints. Analysis (A) includes all 221 prostate cancer patients. while analysis (B) focuses solely on patients with clinically localized disease. MVD was found to offer predictive value in both populations and. in the subpopulation suffering from clinically localized disease. MVD was the only significant ($P$ < 0.0001) predictor of disease-specific survival (Table 2B). By analysing MVD as a dichotomized 'low/high' parameter instead of as a continuous parameter. the relative risk in. for example. the clinically localized cancer patients was 3.84 (95% confidence interval 2.17–6.82) without changing the results of the remaining parameters.

The patients were subjected to watchful waiting and treatment was offered only on occurrence of symptoms. Nearly half (49%) of the patients received endocrine palliative treatment during disease. and the disease-specific survival rates among these patients were significantly ($P$ = 0.04) shorter than among those who did not need palliative treatment. There was a slight majority (55%) of MVD 'high' tumours in the group of palliated patients. while 58% of the tumours in the group of non-palliated patients were categorized as MVD 'low'. However. there remained a significant difference in disease-specific survival between MVD 'high' and MVD 'low' tumour patients. testing the treatment-demanding ($P$ < 0.0001) and the untreated patients ($P$ = 0.0005) separately.

### DISCUSSION

Despite established prognostic criteria in prostate cancer patients. a confident prediction of the clinical outcome in the individual patient is often not possible. This circumstance makes additional prognostic information necessary.

To our knowledge. angiogenesis has not previously been correlated with survival in prostate cancer patients. This study was based on a previously described complete prostate cancer population subjected to watchful waiting (Borre et al. 1997). The present subpopulation represents patients with available archival histological tumour samples obtained at the time of diagnosis as well as complete data records. The follow-up data were almost complete and the cause of death ratio was nearly identical in the current subpopulation and the original population (Table 1). Retrospectively obtained information will never be ideal. and understaging as well as inaccuracy of determination of the cause of death are well-known problems. However. the advantage of retrospective studies is that long-term follow-up is available immediately. Unfortunately. prostate-specific antigen was not available at the time of diagnosis. As the patients were subjected to watchful waiting. endocrine therapy was a surrogate marker for bad prognosis. Therefore. endocrine therapy was not included in the survival models.

 © Cancer Research Campaign 1998

**Figure 2** The microvessel density (MVD) divided into (a) 'low' and (b) 'high' by the median count inside 0.25 mm² tumour 'hot spot' areas correlated with disease-specific survival in (**A**): all 221 patients with prostate cancer irrespective of tumour stage (MVD median 43); (**B**): 125 patients with clinically localized prostate cancer (T1–2, NX, M0; MVD median 37); (**C**): 96 patients with advanced prostate cancer (T > 2 and/or M1; MVD median 53)

**Table 2** Univariate and Cox multivariate analyses of the prognostic value of clinical characteristics and microvessel density for disease-specific survival in (A): 221 prostate cancer patients irrespective of clinical stage, and (B): 125 prostate cancer patients with clinically localized (T1–2, Nx, M0) disease

| Factor | Univariate P-value | Multivariate P-value | RR | CI (95%) |
|---|---|---|---|---|
| A | | | | |
| T-classification[a] | < 0.0001 | < 0.0001 | 1.89 | 1.53–2.32 |
| M-Classification[b] | < 0.0001 | 0.25 | – | – |
| Grade[c] | < 0.0001 | 0.03 | 1.33 | 1.03–1.72 |
| MVD[d] | < 0.0001 | 0.0004 | 1.01 | 1.01–1.02 |
| B | | | | |
| T-classification[e] | 0.03 | 0.06 | – | – |
| Grade[c] | 0.004 | 0.10 | – | – |
| MVD[d] | < 0.0001 | < 0.0001 | 1.03 | 1.02–1.05 |

[a]T1a vs T1b vs T2 vs T > 2. [b]M0 vs M1. [c]Well vs moderate vs poor differentiation. [d]Microvessel density (continuous parameter, area = 0.25 mm²). [e]T1a vs T1b vs T2. RR, relative risk; CI, confidence interval.

Based on the knowledge of the great heterogeneity of prostate cancer (Byar and Mostofi, 1972) and the demonstration of a significantly higher MVD at the centre of the prostate tumour than at the periphery (Siegal et al, 1995), the MVD was quantified as the maximum count in microvessel positive 'hotspot' areas of the tumour.

As a cut-off point representing the median value can be used without introducing bias evaluating prognostic factors (Simon and Altman, 1994) MVD 'low' and 'high' groups were defined by dividing the patients into two equal groups using the median MVD. The survival plots in Figure 2A–C represent three different median values as cut-off points. If, however, the cut-off point of the entire population (median = 43) had been applied to the subpopulations, the significant difference in survival would not have changed, although the distribution of the patients would have been different. In the multivariate analyses (Table 2), MVD was preferred to be analysed as a continuous parameter to retain all the information (Simon and Altman 1994) and the relative risk thereby refers to every single additional microvessel count. However, when analysing MVD as a dichotomized 'low/high' parameter, the relative risk resembled the results found by, for example, Heimann et al (1996) and Fox et al (1994).

Like Weidner et al (1993) who used a larger counting area (0.739 mm²) to estimate the maximal MVD in 79 prostate cancer patients, the current study found a statistically significant association between MVD and increase in both clinical stage and malignancy grade. Weidner et al (1993) found a median MVD per mm² in patients with and without metastases reaching, respectively, 49 and 89 against 160 and 232 in our study (based on the 0.25 mm² counting area).

Counting randomly selected tumour areas by computer, Brawer et al (1994) successfully demonstrated that MVD was a significant predictor of pathological stage in 37 prostate cancer patients. By converting the raw data, measured in the 1.71-mm² area, to vessels per mm², the mean MVD of localized tumours was 80 compared with 110 for tumours with capsular penetration in the same study (Brawer et al, 1994). By a similar conversion of the MVD originally measured in both areas in the current study, the ratios of the mean MVD per mm² between organ-confined and non-organ-confined tumours were nearly identical (about 0.7) to the result of Brawer et al (1994).

© Cancer Research Campaign 1998 

Silberman et al (1997) have demonstrated that MVD correlates with progression after radical prostatectomy in 87 carcinomas. The anti-CD31-immunostained microvessels were quantified in 'hotspot' areas (3.14 mm$^2$). The same study failed to demonstrate a correlation between angiogenesis and pathological stage. However, only intermediate-grade carcinomas were included in the study.

There exist no absolute cut-off numbers for MVD to predict prognosis that have held up across different studies which are in any case difficult to compare directly because of the differences in counting areas. Despite the fact that MVD per mm$^2$ is dependent on the original counting area, we only found marginal differences in the distribution of the patients in MVD 'low' and 'high' categories, using either a 0.25-mm$^2$ or a 0.0625-mm$^2$ area. Although determination of MVD is far from standardized, the current results were based on the MVD determination at ×200 magnification. However, the smallest counting area was slightly superior to the larger when comparing the significance between MVD and clinical outcome in a multivariable analysis. A similar observation, that a small area corresponding to a higher magnification improves the detail of the image, thereby allowing the identification of more single endothelial cell sprouts, has been made in breast cancer (Vermeulen et al, 1996). However, information will be lost if the counting areas do not match the size of the hotspots. As proposed by Vermeulen et al (1996), a standardization of angiogenesis quantification is necessary to facilitate confirmation of the suggested prognostic value of MVD in prospective controlled trials. By standardizing and simplifying the scoring of MVD, it will probably become a useful and important prognostic marker in future. It should be emphasized that the current data are based on material primarily removed by TURP, while the future clinical utility of MVD, together with several other prognostic markers, will be dependent on biopsy techniques, which might turn out to be a critical issue caused by the relative lack of material from this distinctly heterogeneous cancer.

Despite the risk of inaccuracy in data due to retrospectively obtained patient characteristics, as well as immunohistochemical quantification of angiogenesis, the current results of the association between angiogenesis as measured by MVD and survival in patients subjected to watchful waiting suggest that the pattern of neovascularization is important in the natural history of prostate cancer. Angiogenesis, as a predictor of the spontaneous clinical outcome of clinically localized prostate cancer patients, should be included in the decision of future therapeutical strategies of the individual prostate cancer patient.

### ACKNOWLEDGEMENT

This study was supported by grants from The Danish Cancer Society, Clinical Research Unit at Aarhus Oncological Centre.

### REFERENCES

Borre M, Nerstrøm B and Overgaard J (1997) The natural history of prostate cancer based on a Danish population treated with no intent to cure. *Cancer* **80**: 917–928

Borre M, Nerstrøm B and Overgaard J (1998) The dilemma of prostate cancer. A growing human and economic burden irrespective of treatment strategies. *Acta Oncol* **36**: 681–687

Brawer MK, Deering RE, Brown MT, Preston SD and Bigler SA (1994) Predictors of pathologic stage in prostatic carcinoma. *Cancer* **73**: 678–687

Breslow N, Chan CW, Dhom G, Drury RAB, Franks LM, Geillei B, Lee YS, Lundberg S, Sparke B, Sternby NH and Tulinius H (1977) Latent carcinoma of prostate at autopsy in seven areas. *Int J Cancer* **20**: 680–688

Byar DP and Mostofi FK (1972) Carcinoma of the prostate: prognostic evaluation of certain pathologic features in 208 radical prostatectomies. *Cancer* **30**: 5–13

Engeland A, Haldorsen T, Hakulinen T, Hörte LG, Luostarinen T, Magnus K, Schou G, Sigvaldason H, Storm HH, Tulinius H and Vaittinen P (1993) Prediction of cancer incidence in the Nordic countries up to the years 2000 and 2010. *APMIS* **38**: 64–66

Engeland A, Haldorsen S, Tretli S, Hakulinen T, Hörte LG, Luostarinen T, Schou G, Sigvaldason H, Storm HH, Tulinius H and Vaittinen P (1995) Prediction of cancer mortality in the Nordic countries up to the years 2000 and 2010. *APMIS* **49**: 84–86

Folkman J (1990) What is the evidence that tumors are angiogenesis dependent? *J Natl Cancer Inst* **82**: 4–6

Fox SB, Leek RD, Smith K, Hollyer J, Greenall M and Harris AL (1994) Tumor angiogenesis in node-negative breast carcinomas – relationship with epidermal growth factor receptor, estrogen receptor, and survival. *Breast Cancer Res Treat* **29**: 109–116

Heimann R, Ferguson D, Powers C, Recant WM, Weichselbaum RR and Hellman S (1996) Angiogenesis as a predictor of long-term survival for patients with node-negative breast cancer. *J Natl Cancer Inst* **88**: 1764–1769

Hermanek P and Sobin LH (1992) *UICC TNM Classification of Malignant Tumours*. Springer-Verlag: Berlin

Mostofi FK, Sesterhenn IA and Sobin LH (1980) *Histological Typing of Prostate Tumours*. World Health Organization: Geneva

Offersen BV, Borre M and Overgaard J (1998) Immunohistochemical determination of tumor angiogenesis measured by the maximal microvessel density in human prostate cancer. *APMIS* (in press)

Siegal JA, E Yu and Brawer MK (1995) Topography of neovascularity in human prostate carcinoma. *Cancer* **75**: 2545–2551

Silberman MA, Partin AW, Veltri RW and Epstein JI (1997) Tumor angiogenesis correlates with progression after radical prostatectomy but not with pathological stage in Gleason sum 5–7 adenocarcinoma of the prostate. *Cancer* **79**: 772–779

Simon R and Altman DG (1994) Statistical aspects of prognostic factor studies in oncology. *Br J Cancer* **69**: 979–985

Srivastava A, Laidler P, Davies R, Horgan K and Hughes LE (1988) The prognostic significance of tumor vascularity in intermediate-thickness (0.76–4.0 mm thick) skin melanoma. *Am J Pathol* **133**: 419–423

Vermeulen PB, Gasparini G, Fox SB, Toi M, Martin L, McCulloch P, Pezzella F, Viale G, Weidner N, Harris AL and Dirix LY (1996) Quantification of angiogenesis in solid human tumours: an international consensus on the methodology and criteria of evaluation. *Eur J Cancer* **32A**: 2474–2484

Weidner N, Semple JP, Welch WR and Folkman J (1991) Tumor angiogenesis and metastasis in correlation in invasive breast carcinoma. *N Engl J Med* **324**: 1–8

Weidner N, Carroll PR, Flax J, Blumenfeld W and Folkman J (1993) Tumor angiogenesis correlates with metastasis in invasive prostate carcinoma. *Am J Pathol* **143**: 401–409

© Cancer Research Campaign 1998